# Prognostic and clinicopathological significance of cyclin B expression in patients with breast cancer: A meta-analysis

**DOI:** 10.1097/MD.0000000000006860

**Published:** 2017-05-12

**Authors:** Xi Sun, Guangyan Zhangyuan, Liang Shi, Ying Wang, Beicheng Sun, Qiang Ding

**Affiliations:** a Jiangsu Breast Disease Center, the First Affiliated Hospital with Nanjing Medical University; b Liver Transplantation Center of the First Affiliated Hospital, Nanjing Medical University, Nanjing, Jiangsu Province, China.

**Keywords:** breast cancer, clinicopathology, cyclin B, prognosis

## Abstract

**Background:**

Cyclin B plays a crucial role in cancer cell cycle progression and is overexpressed in many human cancers, including breast cancer. However, the prognostic value of cyclin B expression in breast cancer is controversial. We performed a meta-analysis to assess the clinicopathological and prognostic significance of cyclin B expression in breast cancer.

**Methods:**

We searched PubMed, web of science, and Embase databases to retrieve the publications investigating the association between cyclin B expression and clinicopathological/prognostic significance in breast cancer patients. The pooled hazard ratio (HR) or odds ratio (OR) with its 95% confidence intervals (CIs) were used to estimate the effects.

**Results:**

Ten studies with 2366 breast cancer patients were included to evaluate the association between cyclin B expression and overall survival (OS), disease-free survival (DFS), disease-specific survival (DSS), and clinicopathological parameters. The results showed that cyclin B overexpression in breast cancer patients was significantly associated with both poor OS (univariate analysis: HR = 2.38, 95% CI = 1.72–3.30, *P* < .001), DFS (univariate analysis: HR = 1.86, 95% CI = 1.50–2.32, *P* < .001; multivariate analysis: HR = 1.75, 95% CI = 1.22–2.52, *P* = .003), and DSS (multivariate analysis: HR = 5.42, 95% CI = 2.15–13.66, *P* < .001). Additionally, cyclin B overexpression was significantly associated with lymphatic invasion (OR = 2.58, 95% CI = 1.03–6.46, *P* = .017).

**Conclusion:**

Cyclin B overexpression appears to be an independent potential prognostic marker to DSS and DFS for breast cancer. Further studies with large sample size are needed to dissect the relationship between cyclin B and clinicopathological features or prognosis of breast cancer.

## Abstract 2

Supplemental Digital Content is available in the text

## 1 Introduction

As one of the most common cancers worldwide, breast cancer is the leading cause of cancer death among females.^[1]^ With rapid improvements in treatment and early detection, breast cancer death rates decreased by 34% from 1990 to 2010.^[2]^ So far, biomarkers like ER, PR, and HER2 have been found to divide breast cancer into different subtypes and to predict the prognosis of patients.^[3]^ However, intratumor heterogeneity in breast cancer still complicates diagnosis, challenges therapy, and eventually affects patients’ survival.^[4,5]^ So, more reliable biomarkers are required to identify patients at higher risk and to select the most appropriate treatment for an individual patient. Cell cycle checkpoints are critical elements in controlling cell proliferation. Key events in the cell cycle are regulated by the cyclin dependent kinases (CDKs), which are activated by binding specific cyclins.^[6]^ Specific cyclin levels peak at specific times during the cell cycle and produce successive waves of cyclin-CDK activity to regulate the cell cycle events.^[7]^ The cyclin B cluster, which includes cyclin B1 and cyclin B2 in human being, is a subunit of CDK1 and governs the entry into mitosis.^[8–12]^ Cyclin B is synthesized in late S and G2 phases. Cyclin B/CDK1 complex keeps inactive until it is activated by the Cdc25 phosphatase family in prophase, which plays an important role in G2-M phase transition.^[9,12–14]^ Overexpression of cyclin B has proved to drive tumorigenesis in many tumors, including breast cancer.^[15,16]^ Many studies have evaluated the relationship between the expression of cyclin B and survival in breast cancer patients. However, the results of these studies vary from each other, and no consensus has been reached yet. To draw a more precise conclusion, we have therefore undertaken a meta-analysis to assess the role of cyclin B expression as clinicopathological and prognostic molecular marker in breast cancer.

## 2 Material and methods

### 2.1 Search strategy

We searched PubMed, web of science, and Embase databases for articles published up to November 1, 2015 that met the following search criteria: cyclin B OR CCNB AND Breast Neoplasm OR Neoplasm, Breast OR Neoplasms, Breast OR Tumors, Breast OR Breast Tumors OR Breast Tumor OR Tumor, Breast OR Mammary Neoplasms, Human OR Human Mammary Neoplasm OR Human Mammary Neoplasms OR Neoplasm, Human Mammary OR Neoplasms, Human Mammary OR Mammary Neoplasm, Human OR Mammary Carcinoma, Human OR Carcinoma, Human Mammary OR Carcinomas, Human Mammary OR Human Mammary Carcinomas OR Mammary Carcinomas, Human OR Human Mammary Carcinoma OR Breast Cancer OR Cancer, Breast OR Cancer of Breast OR Mammary Cancer OR Malignant Neoplasm of Breast OR Malignant Tumor of Breast OR Breast Carcinoma OR Cancer of the Breast. The studies were limited to human subjects. And there is no language restriction in the literature search. Retrieved papers were independently screened by 2 authors (Sun and Zhang) according to the title, abstract, and type of article, and irrelevant papers were excluded. In addition, the references of identified studies were reviewed to include potentially eligible studies. Systematic review does not involve animal and human experiments, so this article does not require ethical approval.

### 2.2 Inclusion/exclusion criteria

The following criteria were set and reviewed by 2 independent authors (Sun and Zhang): cyclin B expression of breast carcinoma was assessed by immunohistochemistry; articles were published as full paper; odds ratios (ORs) for estimating clinicopathological characteristics were provided or extractable from the original articles; sufficient information was provided to estimate the relation between cyclin B expression and hazard ratios (HRs) of breast neoplasms; when generating HR from published Kaplan–Meier curves, the reported minimum and maximum follow-up times and numbers of people in each arm are needed in these articles; the study reporting the largest dataset was included if several publications reported data from overlapping samples; and only retrospective or prospective cohort studies were included.

### 2.3 Data extraction

Two authors (Sun and Zhang) independently extracted the following data: first author, publication year, country, sample size, cut-off values, clinicopathological and prognostic characteristics, duration of follow-up, and other relevant data. Disagreements between reviewers were resolved by discussion.

### 2.4 Quality assessment

Two authors (Sun and Zhang) independently evaluated the methodological quality of all included studies using Newcastle-Ottawa scale (NOS),^[17]^ and the discrepancy was resolved by discussion. Each study in this meta-analysis was categorized with the NOS system, which is comprised of 3 dimensions (selection of cohort, comparability of cohort, and ascertainment of outcome). The NOS, a star system, ranges from 0 to 9 stars, with more stars indicating a better quality. All of the included studies were awarded 7 or 8 stars in total.

### 2.5 Statistical analysis

The ORs with corresponding 95% confidence intervals (CIs) were used to assess the relationship between cyclin B expression and clinicopathological characteristics in patients with breast neoplasms. The clinicopathological characteristics extracted by meta-analysis included: the presence or absence of lymphatic invasion; clinical stage I and stage II versus stage III and stage IV; tumor grade 1 and grade 2 versus grade 3 and grade 4; tumor size larger than 2 cm versus less than 2 cm; age more than 50 versus less than 50 years; ER positive or negative; PR positive or negative; and HER-2 positive or negative. For prognosis, we used the HRs with corresponding 95% CIs to estimate the effect of cyclin B expression on survival rates. We directly extracted HRs with corresponding 95% CIs if they were provided in the original articles. Otherwise, the methods described by Parmar et al^[18]^ and Tierney et al^[19]^ were used to calculate the data. Kaplan–Meier curves were read using Engauge Digitizer version 4.1 (http://digitizer.sourceforge.net/). The survival data read from Kaplan–Meier curves were entered in the spreadsheet based on Tierney when extracting the HR from published Kaplan–Meier curves, the reported minimum and maximum follow-up times and the reported numbers at risk are needed. Chi-square test and inconsistency index *I*^2^ were utilized to assess or quantify the heterogeneity of included studies. If *P* > .10 and *I*^2^ < 50%, we employed the fixed-effect model, otherwise, a random effects model was used where *P* < .10 or *I*^2^ > 50%. In order to assess the publication bias, funnel plots were used to detect underlying publication bias, with the plots’ asymmetry being estimated by Begger test.^[20]^ Data management and analysis were performed with STATA 12.0 software (Stata Co., College Station, TX).

## 3 Results

### 3.1 Search results

**Figure 1:**
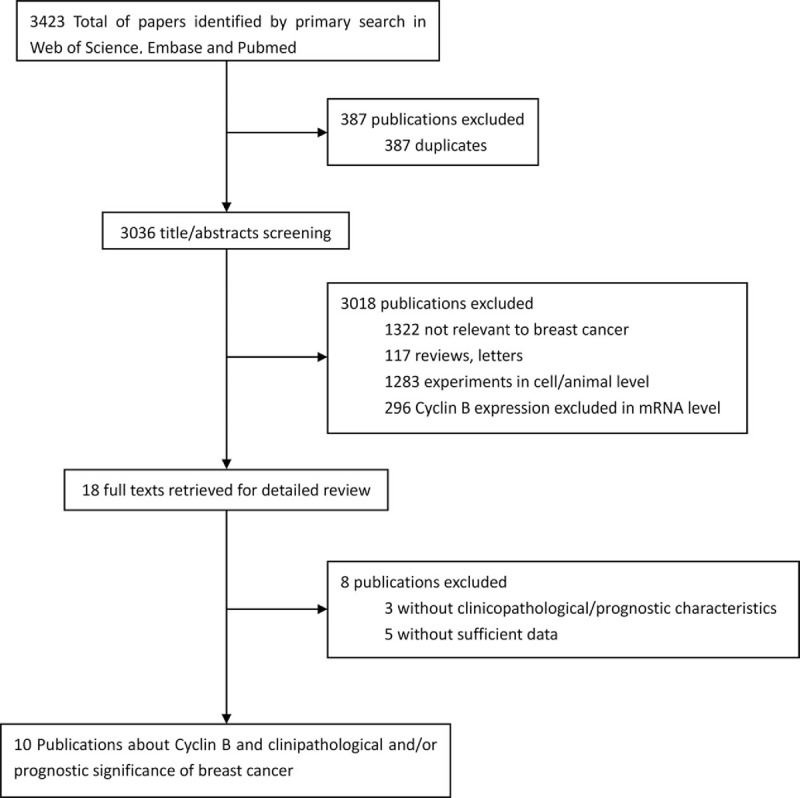
Flow diagram of publications searching and screening process.

As shown in Fig. 1, 3423 studies were initially retrieved from the databases including web of science, Embase, and Pubmed, of which 387 were excluded because of duplicates. After screening the titles and abstracts, 3018 publications were excluded according to inclusion criteria. Of the remaining 18 candidate articles, 3 publications provide cyclin B expression only and 5 publications, analyzing the relationship between cyclin B expression and clinicopathological/prognostic significance, could not be extracted with sufficient information. Ten eligible publications^[21–30]^ were therefore included in the meta-analysis.

### 3.2 Study characteristics

The detailed information of each study is summarized in Table 1. Ten studies, designed as retrospective cohort, investigated a total of 2366 cases from China, Sweden, Germany, Korea, and Japan. Immunohistochemistry was utilized to assess cyclin B expression in all studies. Of the 10 studies included in this meta-analysis, 6 studies^[21,24,25,27–29]^ investigated the association between cyclin B expression and prognostic significance, 2 studies^[23,30]^ evaluated its clinicopathological parameters in patients with breast cancer, and 2 publications^[22,26]^ studied both. In these 10 studies, 3 publications^[24,28,29]^ investigated the total cyclin B expression while 1 publication^[22]^ studied the cyclin B2 expression and 6 publications^[21,23,25–27,30]^ studied the cyclin B1 expression.

**Table 1:**
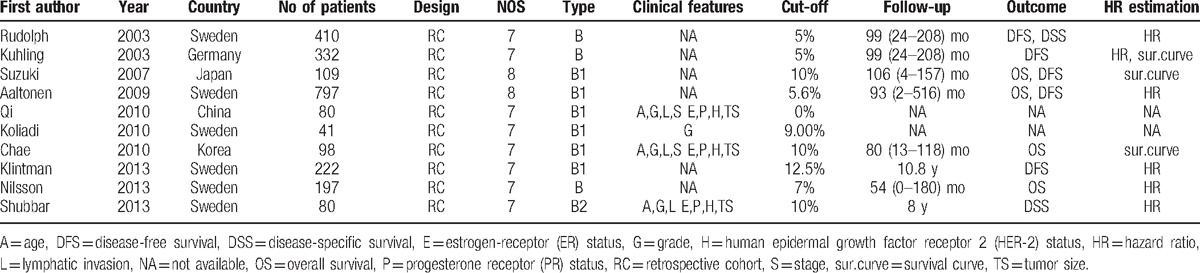
Clinicopathological and methodological features of eligible studies.

### 3.3 Impact of cyclin B expression on survival rates of patients with breast cancer

**Figure 2:**
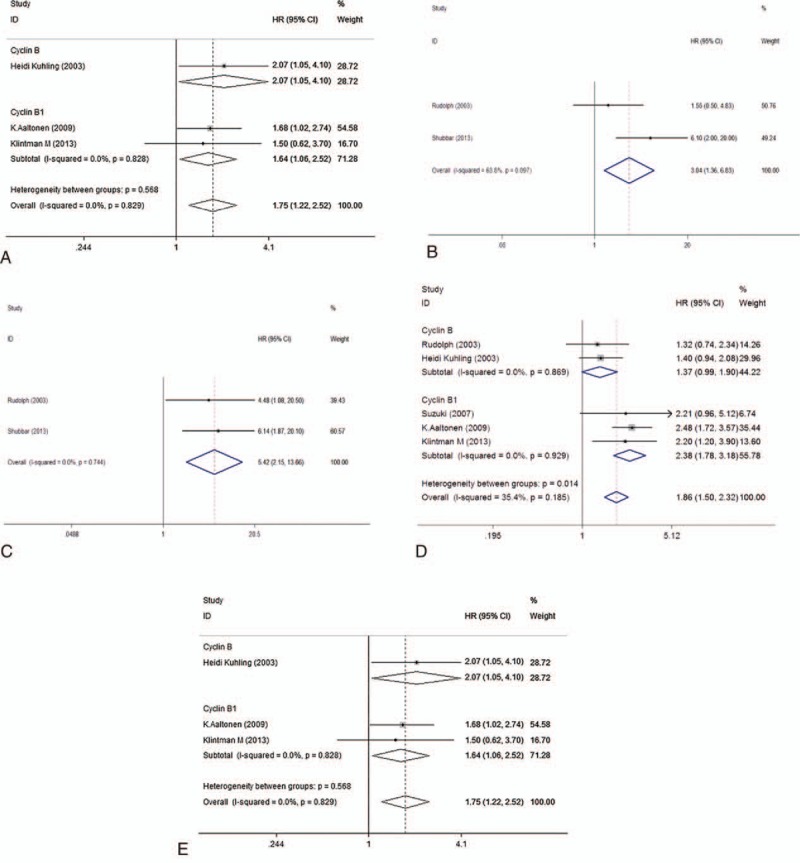
Forest plot of the hazard ratio (HR) for the association of cyclin B expression with prognosis. (A) Overall survival in univariate analysis, (B) disease-specific survival in univariate analysis. (C) Disease-specific survival in multivariate analysis, (D) disease-free survival in univariate analysis. (E) Disease-free survival in multivariate analysis.

**Figure 3:**
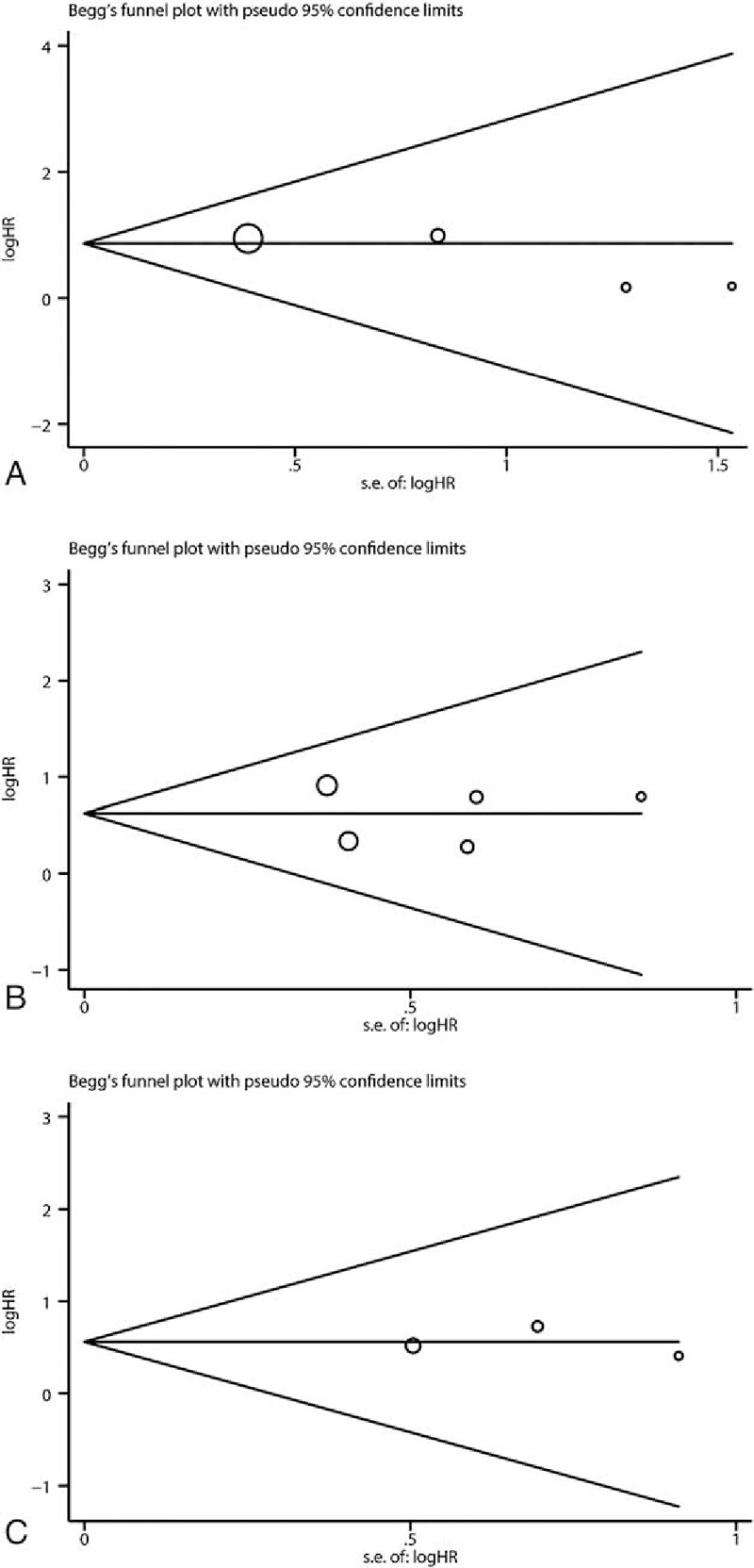
Funnel plots assessing potential publication bias for prognosis. (A) Overall survival in univariate analysis, (B) disease-free survival in univariate analysis. (C) Disease-free survival in multivariate analysis.

**Figure 4:**
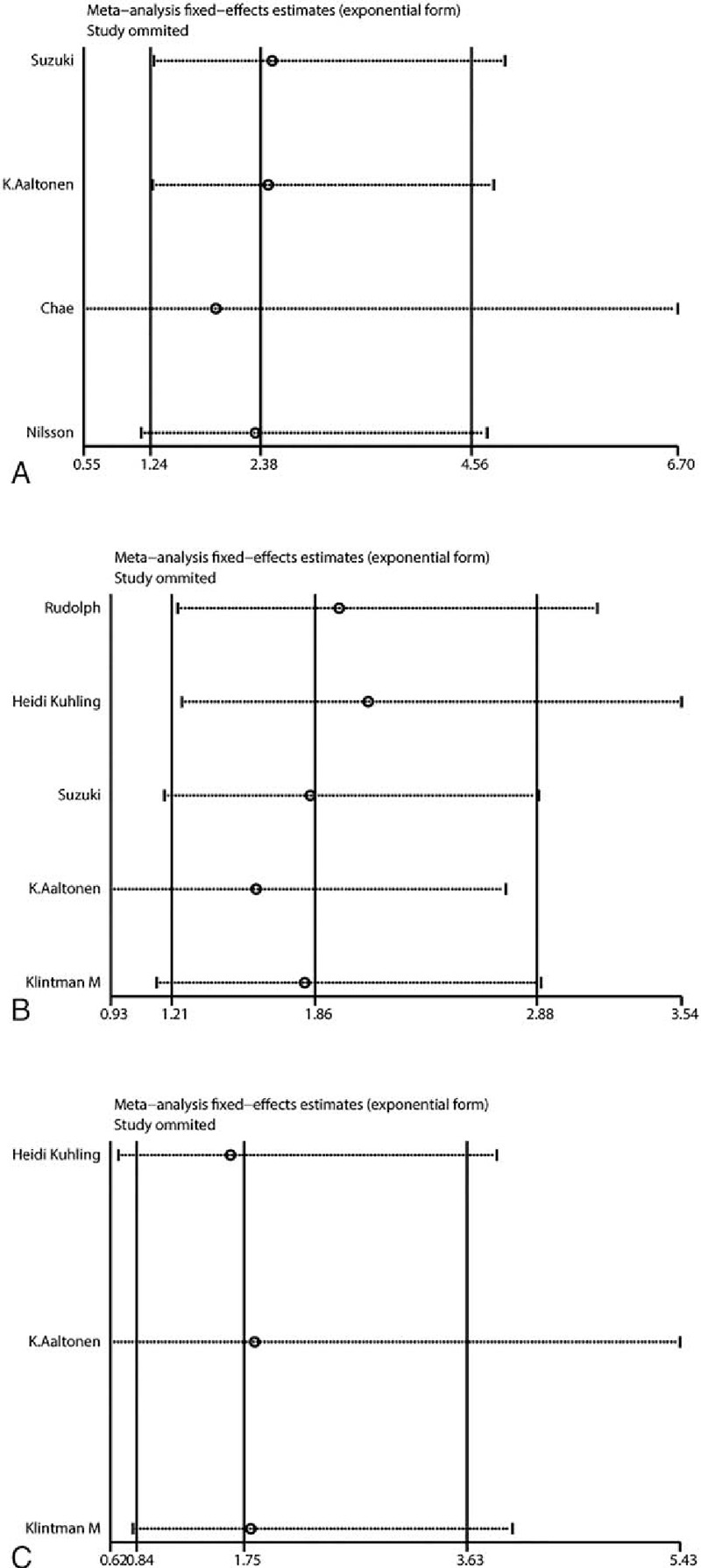
Sensitivity analysis based on stepwise omitting 1 study at a time for prognosis. (A) Overall survival in univariate analysis, (B) disease-free survival in univariate analysis, and (C) disease-free survival in multivariate analysis.

As shown in Table 2 and Fig. 2, meta-analysis of 4 studies^[21,26–28]^ on the prognostic value of cyclin B expression indicated that high level of cyclin B was associated with poor overall survival (OS, univariate analysis:HR = 2.38, 95% CI = 1.72–3.30), without significant heterogeneity between studies (*I*^2^ = 0%, *P* = .53). Five studies^[21,24,25,27,29]^ and 3 studies^[24,25,27]^ assessed the relationship between cyclin B expression and disease-free survival (DFS) in univariate and multivariate analysis, respectively. Both combined HRs suggest that high level of cyclin B expression is an indicator of poor DFS (univariate analysis: HR = 1.86, 95% CI = 1.50–2.32, *P* < .001, multivariate analysis: HR = 1.75, 95% CI = 1.22–2.52, *P* = .003). No significant heterogeneity between studies was detected (univariate analysis: *I*^2^ = 35.4%, *P* = .185, multivariate analysis: *I*^2^ = 0%, *P* = .829). As to the association between cyclin B expression and disease-specific survival (DSS), while no statistical significance was detected in pooled HR of 2 studies^[22,29]^ in univariate analysis (HR = 3.06, 95% CI = 0.80–11.73, *P* = .102) with heterogeneity (*I*^2^ = 68.3%, *P* = .097), the result in multivariate analysis suggested that high cyclin B expression was associated with poor DSS in breast cancer patients (HR = 5.42, 95% CI = 2.15–13.66, *P* < .001), without heterogeneity (*I*^2^ = 0%, *P* = .744). Subgroup analysis was done to differentiate the effects between total cyclin B and its subtype when number of studies is at least 3. To test the robustness of association between cyclin B expression and survival outcome (OS and DFS), potential publication bias and sensitivity were assessed using Begg funnel plot and sensitivity analysis. As shown in Fig. 3, Begg test (*P*_uni-OS_ = .308, *P*_uni-DFS_ = 1.000, and *P*_multi-DFS_ = 1.000) demonstrated no obvious publication bias in this meta-analysis of prognosis. Sensitivity analysis indicated that no significant variation was detected in combined HR by excluding any of the study, confirming the stability of final results (Fig. 4).

**Table 2:**
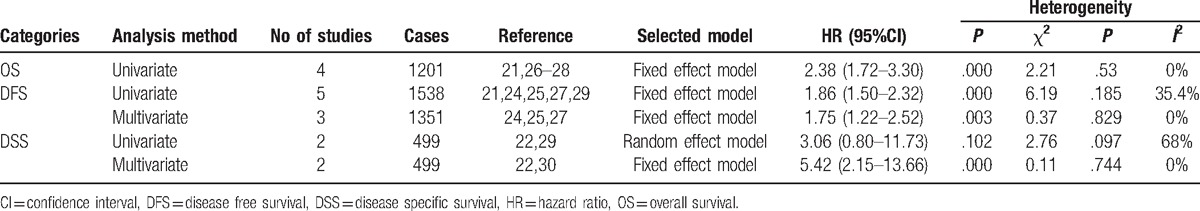
Meta-analysis estimating cyclin B with prognosis.

### 3.4 Association between cyclin B expression and clinicopathologic parameters

Four studies^[22,23,26,30]^ assessed the association between cyclin B expression and clinicopathological parameters including age, tumor size, tumor stage, tumor grade and lymphatic invasion, ER, PR, or HER-2 status (Table 3). Pooled data suggested a significant relationship between cyclin B expression and lymphatic invasion (OR = 2.58, 95% CI = 1.03–6.46) (see Fig. 1, Supplemental Content). However, no significant associations were found between cyclin B expression and age, tumor size, tumor stage, tumor grade, ER, PR, or HER-2 status (see Figs. 2–8, Supplemental Content).

**Table 3:**
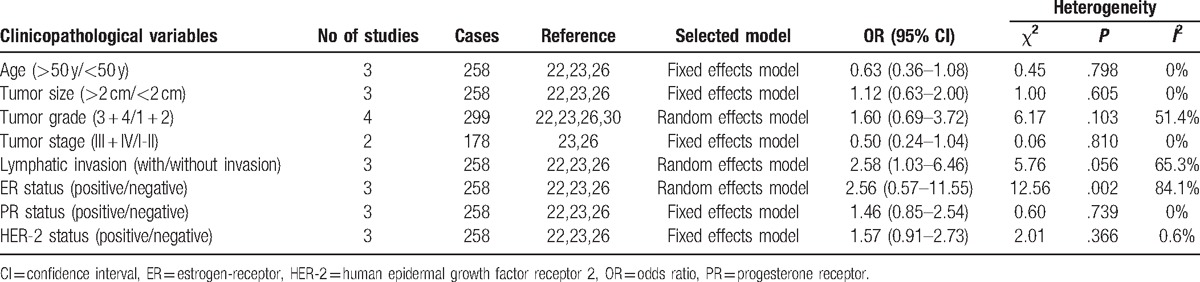
Meta-analysis estimating the relevance of cyclin B with clinicopathological characteristics.

## 4 Discussion

The cyclin B/CDK1 complex governs the G2-M phase transition and is essential for the initiation of mitosis.^[10]^ A negligent G2/M checkpoint will cause genomic instability and induce cancer genesis. So, aberrant cyclin B expression causes uncontrolled cell growth and promotes malignant transformation.^[21,31]^ The overexpression of cyclin B has been shown to be an important factor affecting survival in several malignant diseases, including breast cancer,^[26]^ esophageal squamous cell carcinoma,^[32]^ nonsmall cell carcinoma,^[33]^ and hepatocellular carcinoma.^[34]^ Several studies with controversial results have been done to study the relationship between cyclin B and survival. This meta-analysis has drawn a preciser conclusion about this topic.

To our knowledge, this is the first meta-analysis about the relationship between cyclin B and DSS, OS, DFS, and clincopathological parameters in breast cancer. In this meta-analysis, we included 10 eligible articles about patients with breast cancer to draw a conclusion that overexpression of cyclin B is significantly associated with poor DSS and DFS in breast cancer patients, indicating that cyclin B may be a promising molecular marker in this disease. Two studies^[22,29]^ were included when analyzing prognostic influence of cyclin B on DSS of breast cancer. In univariate analysis, the prognostic value of cyclin B was not confirmed by combining data. Low heterogeneity was found between the 2 studies. Due to the number of the studies, the source of heterogeneity was not able to evaluate and more studies are needed to study the relationship between univariate DSS and cyclin B. Only 1 study^[27]^ reported the HR (1.83,95% CI = 0.99–3.40, *P* = .05) in multivariate analysis of OS, and more studies are required to assess the prognostic role of cyclin B in OS in multivariate analysis. We further demonstrated that cyclin B is correlated with the presence of lymph node metastasis, indicating that patients with overexpression of cyclin B are prone to have lymphatic invasion. Some studies^[21,27,28]^ investigated the relationship between cyclin B and clincopathological coefficient using the correlation parameters which prevented us from pooling these data to combine. In addition, the multivariable analyses of different studies did not control for the same covariates, which is a source of heterogeneity and more studies are needed to clarify this. Cyclin B1 and cyclin B2, which share the same binding motif, contain a 100-amino-acid region of sequence similarity to the consensus “cyclin box” and both bind to CDK1 to form the cyclin B/cdk1 complex, which phosphorylates a critical set of proteins to set into motion the events that define mitosis when activated.^[35]^ On account of the similar function of cyclin B1 and B2 in regulating cell cycle, 3 studies^[24,28,29]^ detected total cyclin B expression including both B1 and B2 while other studies detected either 1 of the 2 cyclins. Subgroup analysis showed both total cyclin B and cyclin B1 play an important role in predicting poor prognosis in OS with univariate analysis and in DFS with multivariate analysis.

Nuclear translocation of cyclin B plays an essential role in promoting mitosis.^[36]^ Cyclin B/Cdc2 is cytoplasmic during interphase and is transported into the nucleus at the beginning of mitosis.^[37,38]^ Suzuki et al^[21]^ concluded that only nuclear cyclin B acts as prognostic factor in breast cancer. Winters et al^[15]^ found that both nuclear and cytoplasmic cyclin B were significant predictors of poor prognosis in breast cancer. In this meta-analysis, we included studies without differentiating between nuclear and cytoplasmic expression. Inadequate data extracted from these studies make it unable to combine data both in cytoplasm and nuclear. More studies are needed to investigate the effect of cyclin B location on the prognosis of breast cancer patients.

In conclusion, this meta-analysis draws a preciser conclusion that there are significant associations between cyclin B overexpression and poor survival in patients with breast cancer, indicating that cyclin B may be a potential biomarker in breast cancer. To strengthen our findings, well-designed prospective studies with lager number of cases should help to explore the relationship between cyclin B overexpression and survival of breast cancer.

## Acknowledgments

The authors thank to the authors of the primary studies.

## Supplementary Material

Supplemental Digital Content
