# Supplementary material for: Prognostic and clinicopathological significance of cyclin B expression in patients with breast cancer: A meta-analysis
Source: Medicine (Baltimore). 2017 May 12;96(19):e6860. doi: 10.1097/MD.0000000000006860 (PMC5428614; doi:10.1097/MD.0000000000006860)

Supplementary Content

**Supplementary Figure 1 Forest plot of the odds ratio (OR) for the association of cyclin B expression with lymphatic invasion.**


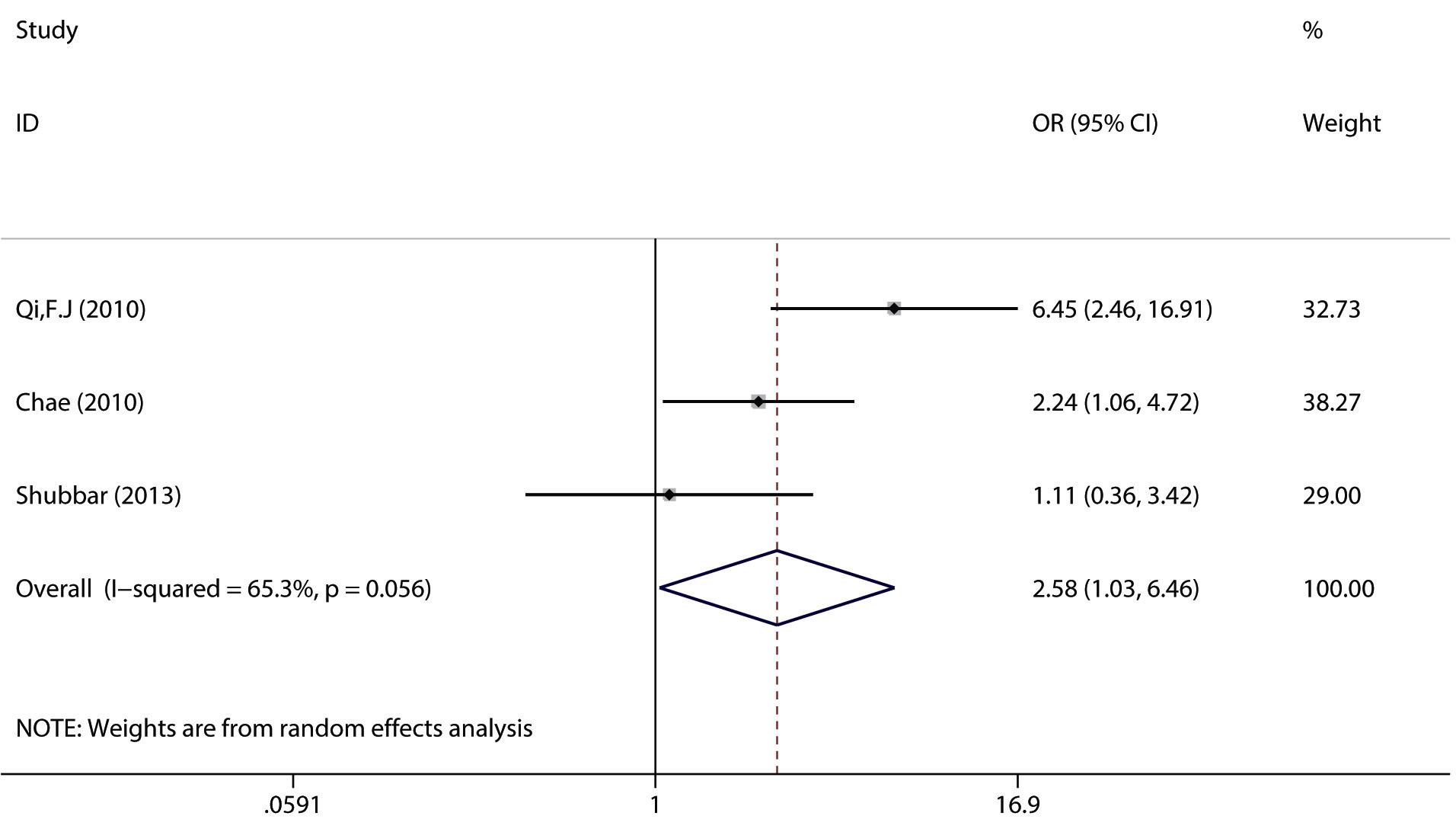


**Supplementary Figure 2Forest plot of the odds ratio (OR) for the association of cyclin B expression with tumor size.**


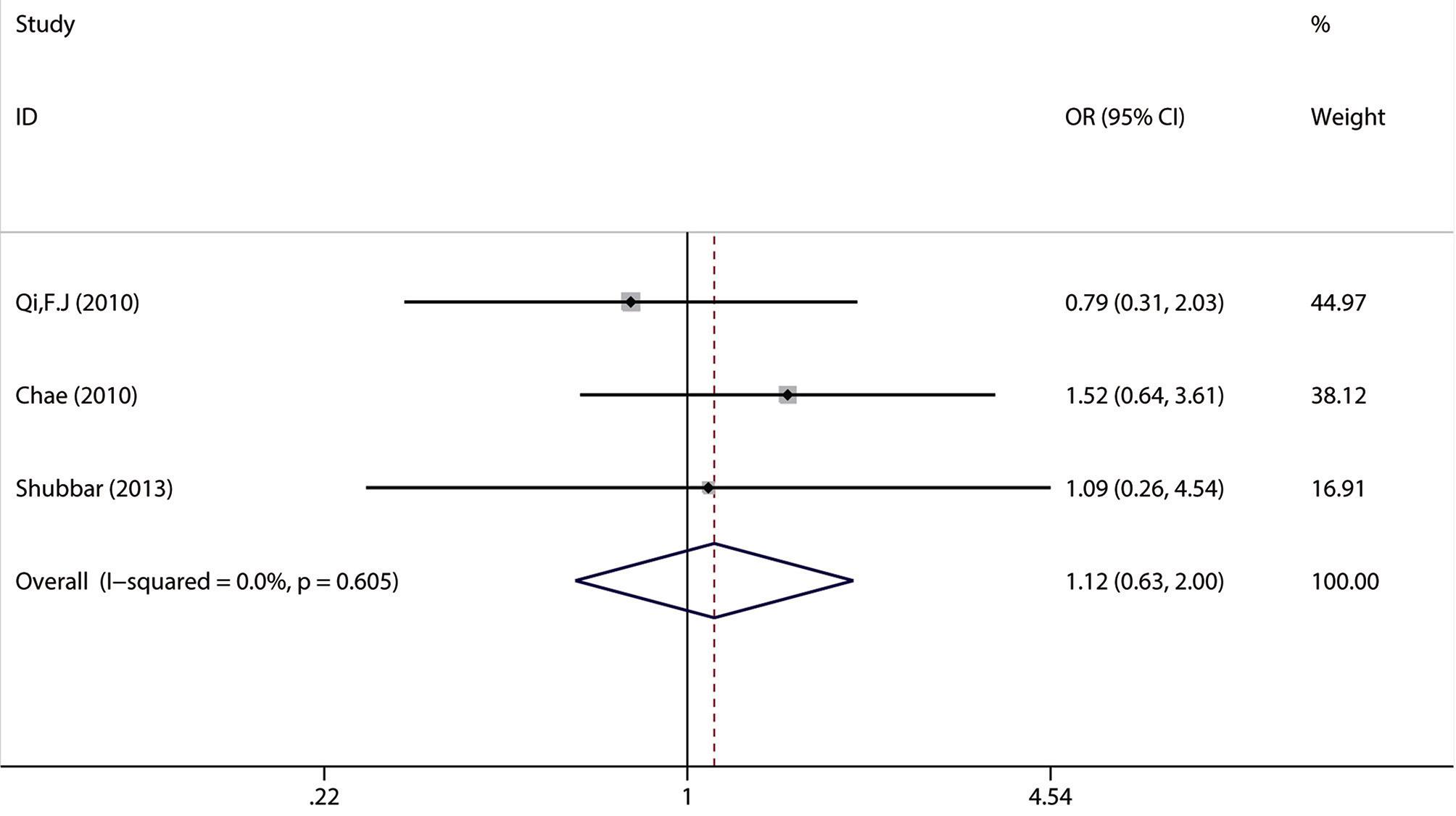


**Supplementary Figure 3 Forest plot of the odds ratio (OR) for the association of cyclin B expression with stage.**


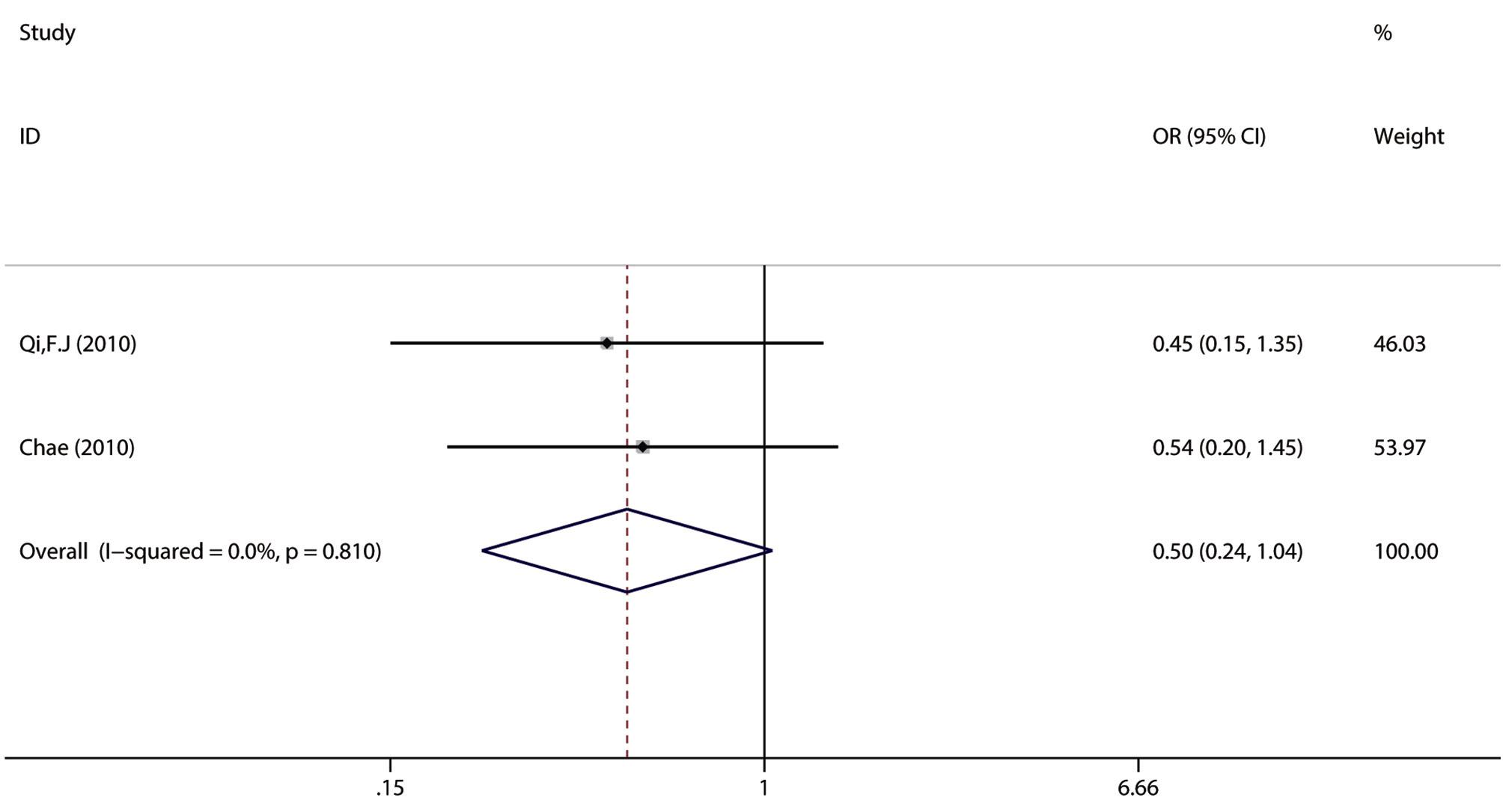


**Supplementary Figure 4 Forest plot of the odds ratio (OR) for the association of cyclin B expression with grade.**


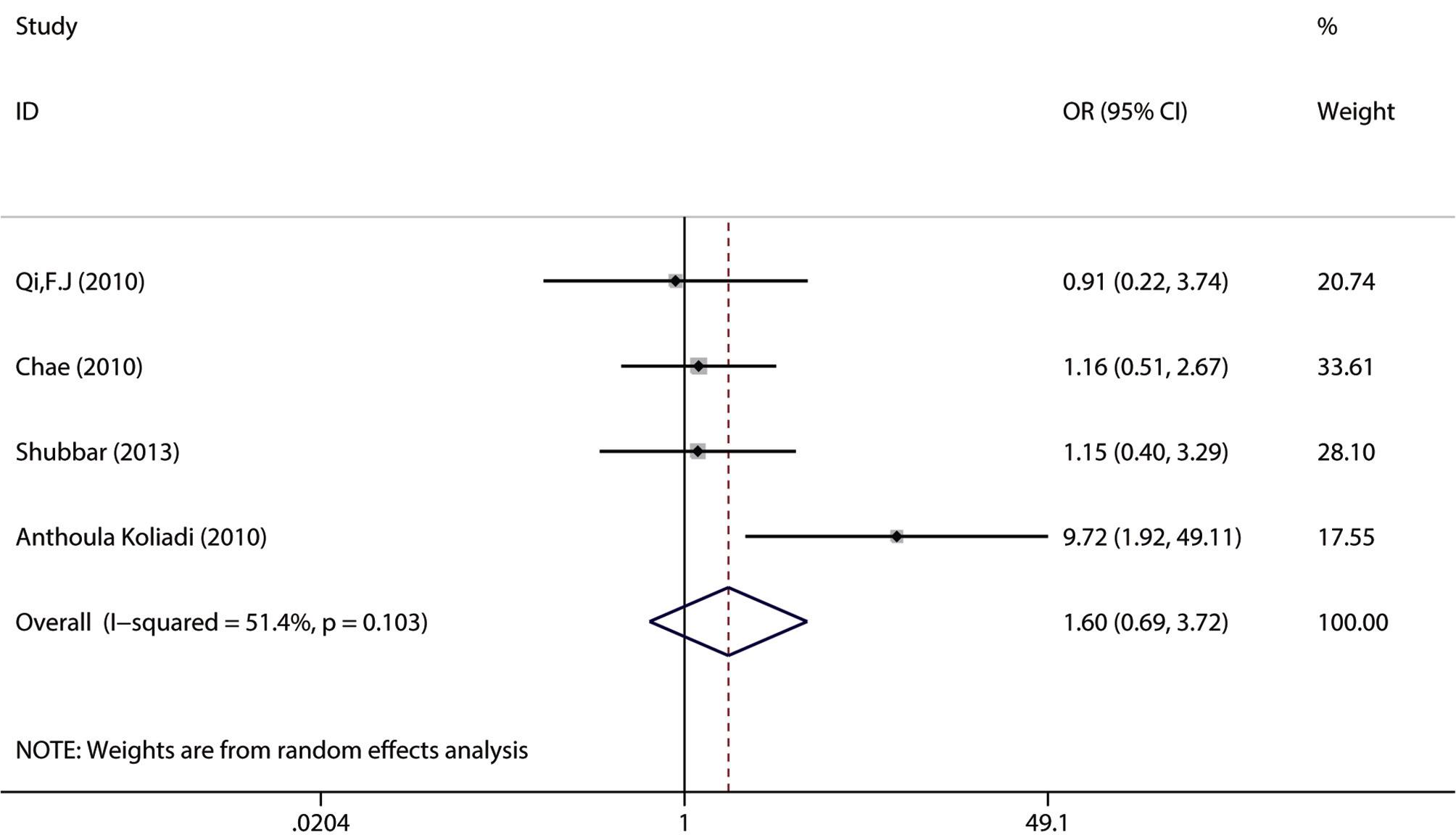


**Supplementary Figure 5 Forest plot of the odds ratio (OR) for the association of cyclin B expression with age.**


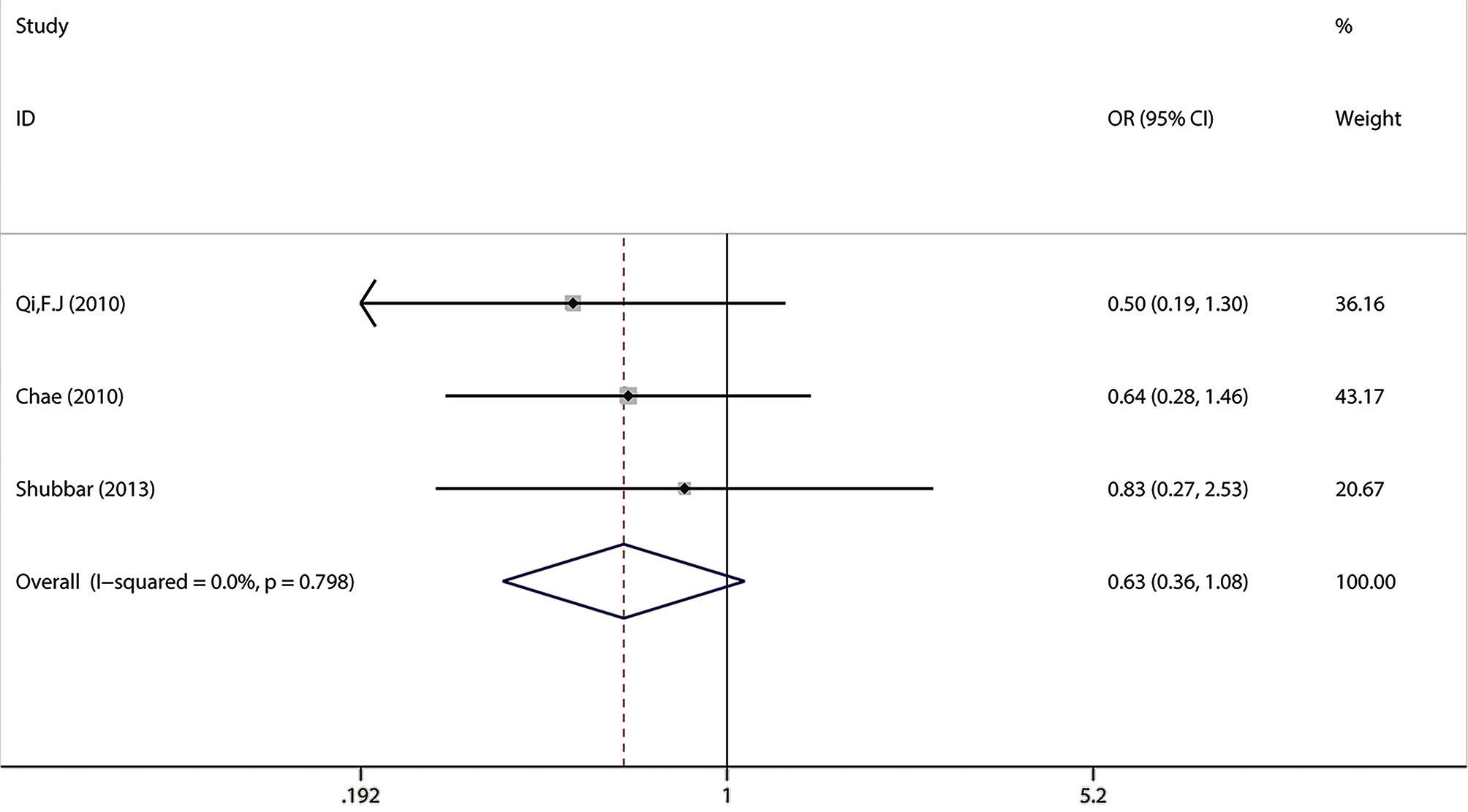


**Supplementary Figure 6 Forest plot of the odds ratio (OR) for the association of cyclin B expression with ER status.**


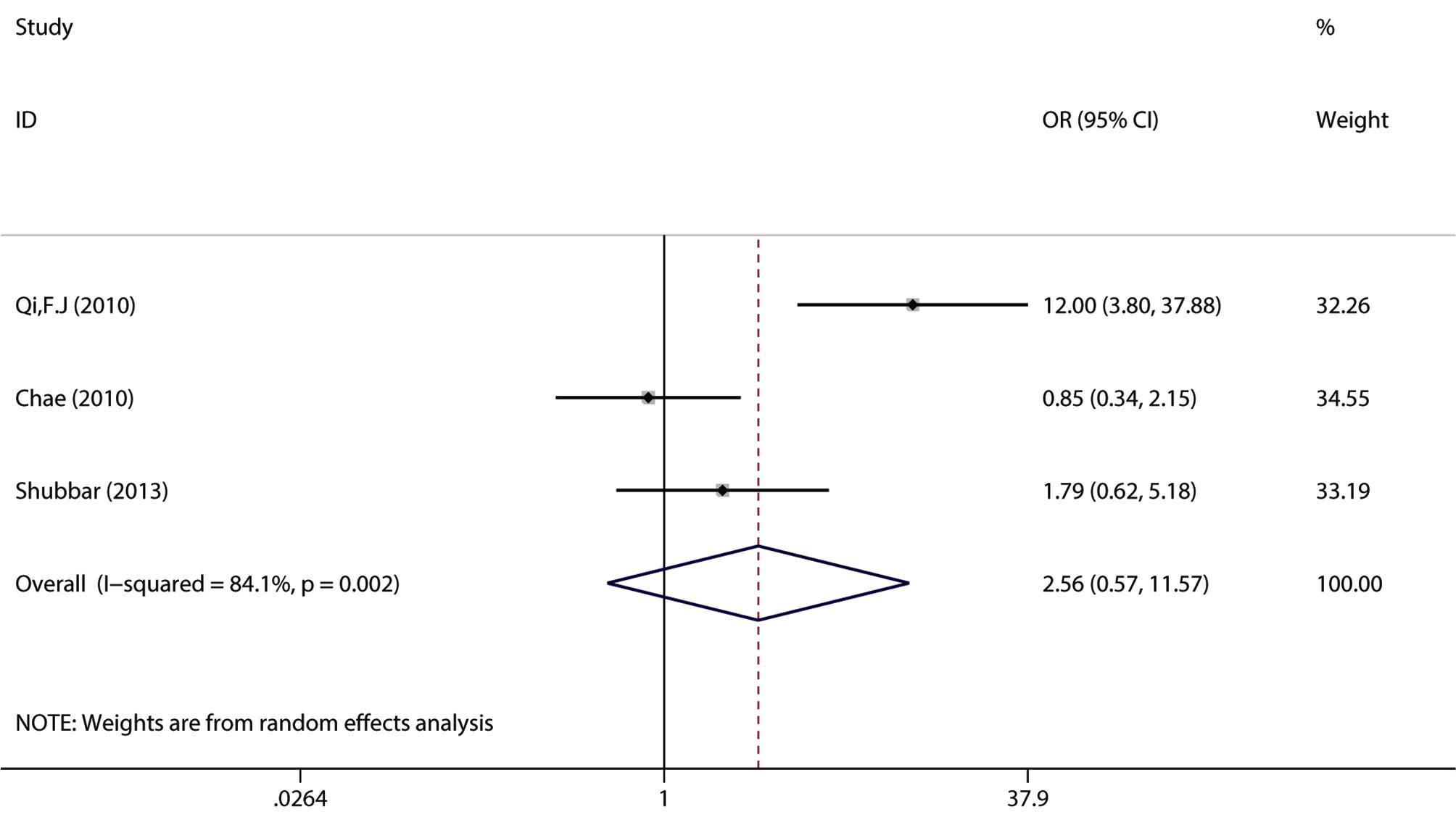


**Supplementary Figure 7 Forest plot of the odds ratio (OR) for the association of cyclin B expression with PR status.**


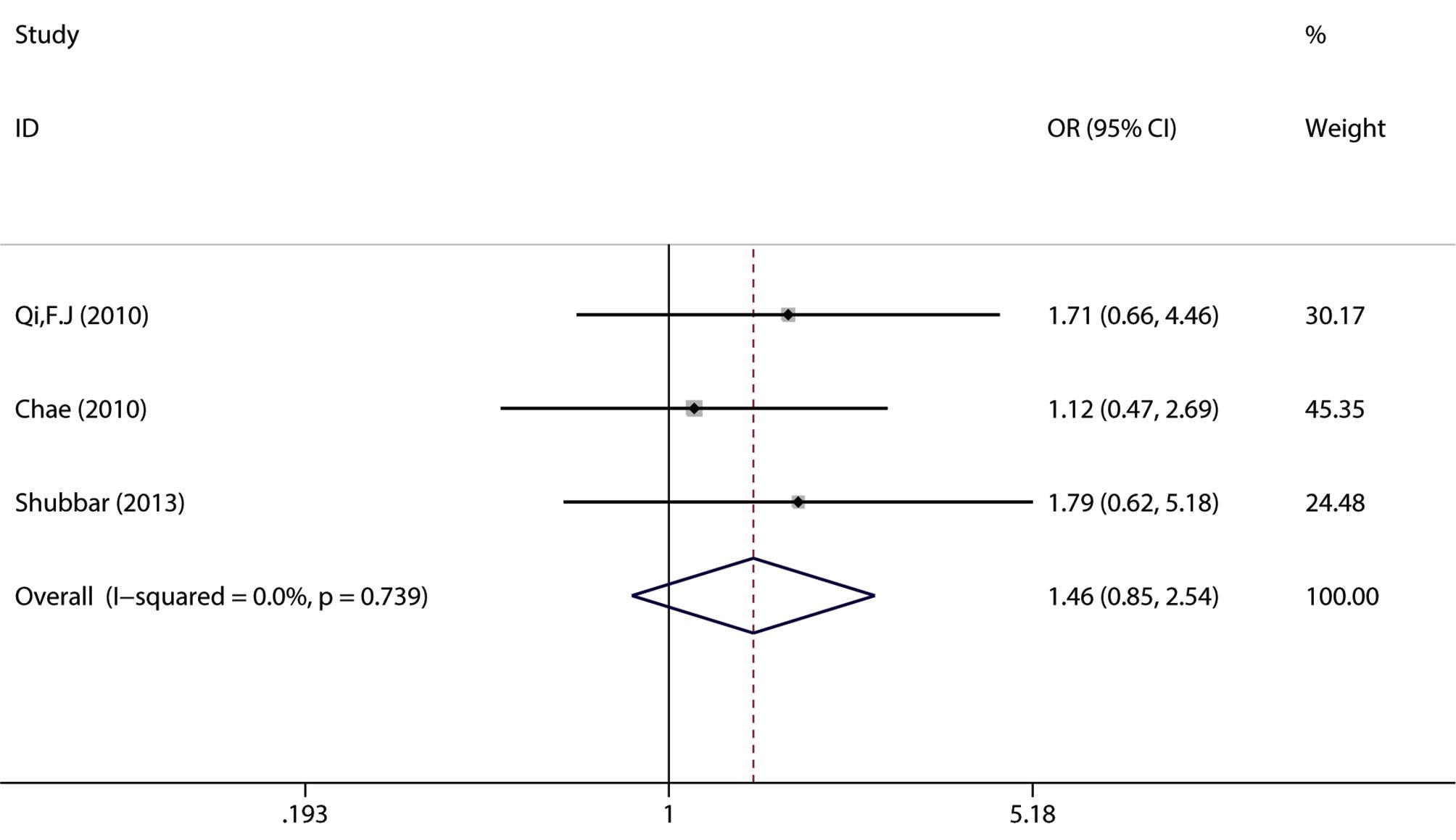


**Supplementary Figure 8 Forest plot of the odds ratio (OR) for the association of cyclin B expression with HER-2 status.**


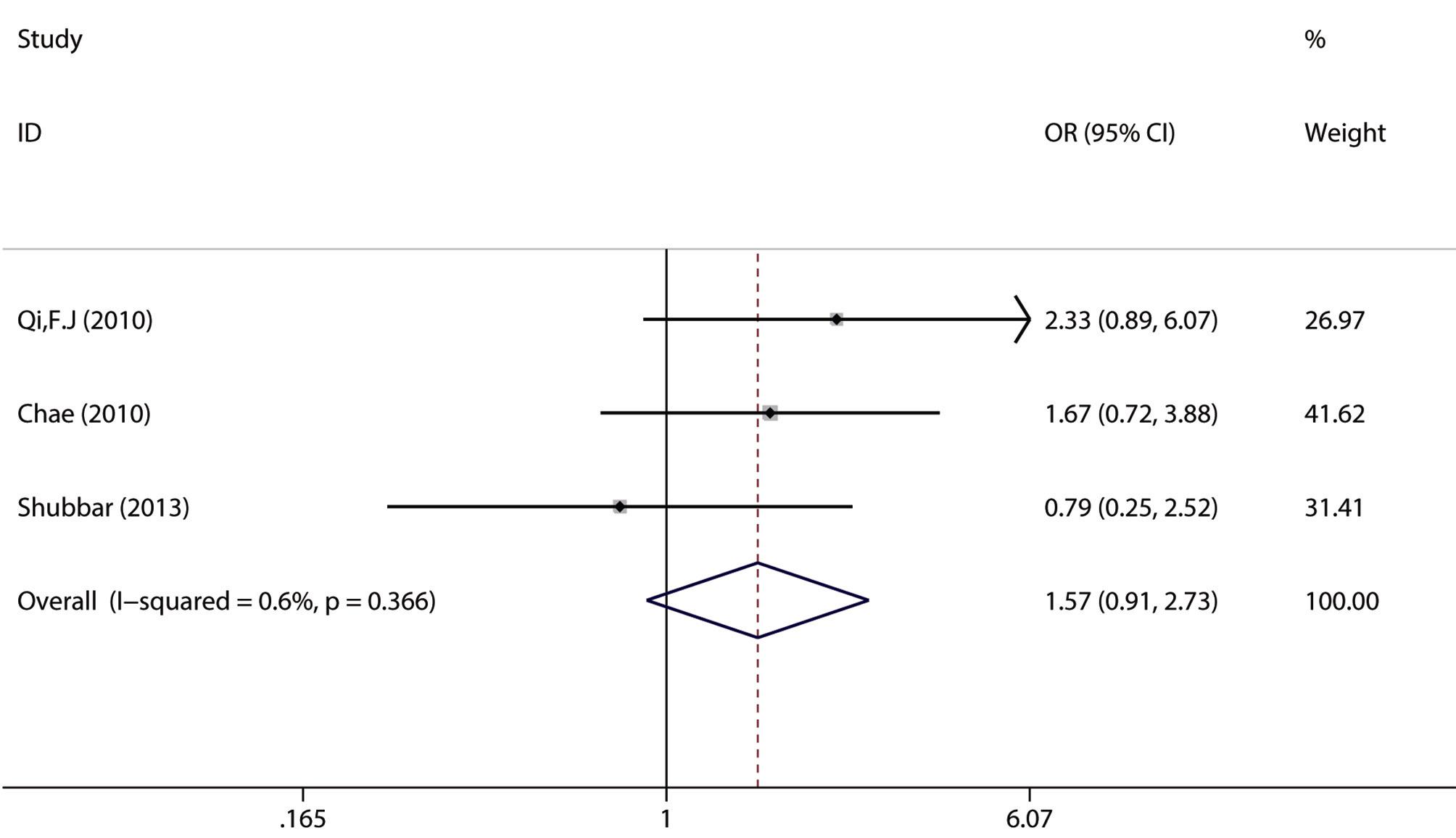

Supplement: Supplemental Digital Content [file medi-96-e6860-s001.doc]
